# Advances in targeting myocardial fibrosis: integrating mechanisms and therapeutics

**DOI:** 10.3389/fcvm.2026.1769016

**Published:** 2026-03-04

**Authors:** Zihui Xu, Yuyan Zhao

**Affiliations:** The First Affiliated Hospital of China Medical University, Shenyang, Liaoning, China

**Keywords:** drug development, fibroblast activation, heart failure, myocardial fibrosis, therapeutic targets

## Abstract

Myocardial fibrosis (MF) is a maladaptive pathological response of the heart to chronic injury. Accumulating evidence indicates that MF plays a central role in the development and progression of hypertensive heart disease, ischemic cardiomyopathy, diabetic cardiomyopathy, and heart failure, and is closely associated with an increased risk of arrhythmias and sudden cardiac death. In recent years, advances in experimental and analytical approaches have improved our understanding of the molecular mechanisms underlying MF and informed the development of potential therapeutic strategies. However, many existing pharmacological interventions exhibit limited target specificity, uncertain long-term efficacy, and incompletely defined mechanisms of action in humans. In this review, we summarize the major molecular pathways involved in myocardial fibrosis and discuss current and emerging therapeutic approaches, incorporating mechanistic insights from recent single-cell and spatial transcriptomic studies to better contextualize fibrotic signaling heterogeneity and translational challenges.

## 1 Background

Cardiovascular diseases (CVDs) remain the leading cause of morbidity and mortality worldwide, accounting for more than one-third of global deaths (1, 2). With population aging and the increasing prevalence of cardiometabolic disorders such as hypertension, diabetes, obesity, and chronic kidney disease, the burden of structural heart disease continues to increase. Across diverse etiologies, myocardial fibrosis (MF) has emerged as a common and pivotal pathological substrate underlying adverse cardiac remodeling (3, 4).

Epidemiological studies indicate that myocardial fibrosis is highly prevalent among patients with cardiometabolic diseases and heart failure, affecting approximately one-third of these populations (4), with an even higher prevalence in individuals with long-standing hypertension, ischemic heart disease, or diabetic cardiomyopathy. Importantly, MF is not merely a histopathological finding but is strongly associated with diastolic dysfunction, ventricular arrhythmias, sudden cardiac death, and progression to heart failure (HF) with both preserved and reduced ejection fraction (3).

Advances in noninvasive imaging modalities (5–7), including cardiac magnetic resonance–derived late gadolinium enhancement and extracellular volume quantification, as well as emerging circulating biomarkers (8), have enabled earlier detection and risk stratification of myocardial fibrosis. However, despite advances in diagnosis and risk stratification, effective therapies that directly and specifically target fibrotic remodeling remain limited. Current guideline-directed medical therapies for heart failure, such as renin–angiotensin–aldosterone system (RAAS) inhibitors (9) and sodium–glucose cotransporter 2 (SGLT2) inhibitors (10), exert indirect antifibrotic effects. However, their target specificity is low, and their long-term capacity to reverse established fibrosis is uncertain.

At the mechanistic level, myocardial fibrosis results from the coordinated activation of multiple profibrotic pathways, including mechanical stress–induced signaling, neurohumoral activation, metabolic dysregulation (11), inflammation (12), oxidative stress (OS) (13, 14), and extracellular matrix (ECM) remodeling, ultimately converging on cardiac fibroblast activation and myofibroblast differentiation. A major challenge in the field is the pronounced heterogeneity of fibrotic responses across disease contexts, stages, and myocardial regions, which complicates target identification and therapeutic translation.

Together, these observations highlight the need for a comprehensive and mechanistically grounded understanding of myocardial fibrosis to inform therapeutic development. In this review, we synthesize current evidence on the molecular mechanisms underlying myocardial fibrosis and critically evaluate existing and emerging therapeutic strategies, with a focus on translational relevance and future research directions.

## 2 Molecular mechanisms of myocardial fibrosis

Myocardial fibrosis is driven by a complex and highly interconnected network of molecular mechanisms involving mechanical stress–induced signaling, neurohumoral activation, metabolic dysregulation, inflammatory pathways, oxidative stress, non-coding RNA regulation, and extracellular matrix remodeling. Although these mechanisms are often described as discrete profibrotic axes, they do not operate in isolation. Instead, they interact and converge to regulate cardiac fibroblast activation, myofibroblast differentiation, and excessive ECM deposition, ultimately leading to increased myocardial stiffness and impaired cardiac function.

Importantly, the activation and functional impact of these profibrotic pathways are not uniform but vary according to disease context, stage of injury, and myocardial microenvironment. Recent advances in single-cell RNA sequencing (15) and spatial transcriptomics (16) have provided a conceptual framework for understanding this heterogeneity by revealing that fibrotic signaling pathways are engaged in a cell-state and spatially restricted manner, rather than being uniformly activated across the myocardium. These insights help explain the variable phenotypic manifestations of myocardial fibrosis and the inconsistent therapeutic responses observed in clinical practice.

**Figure 1:**
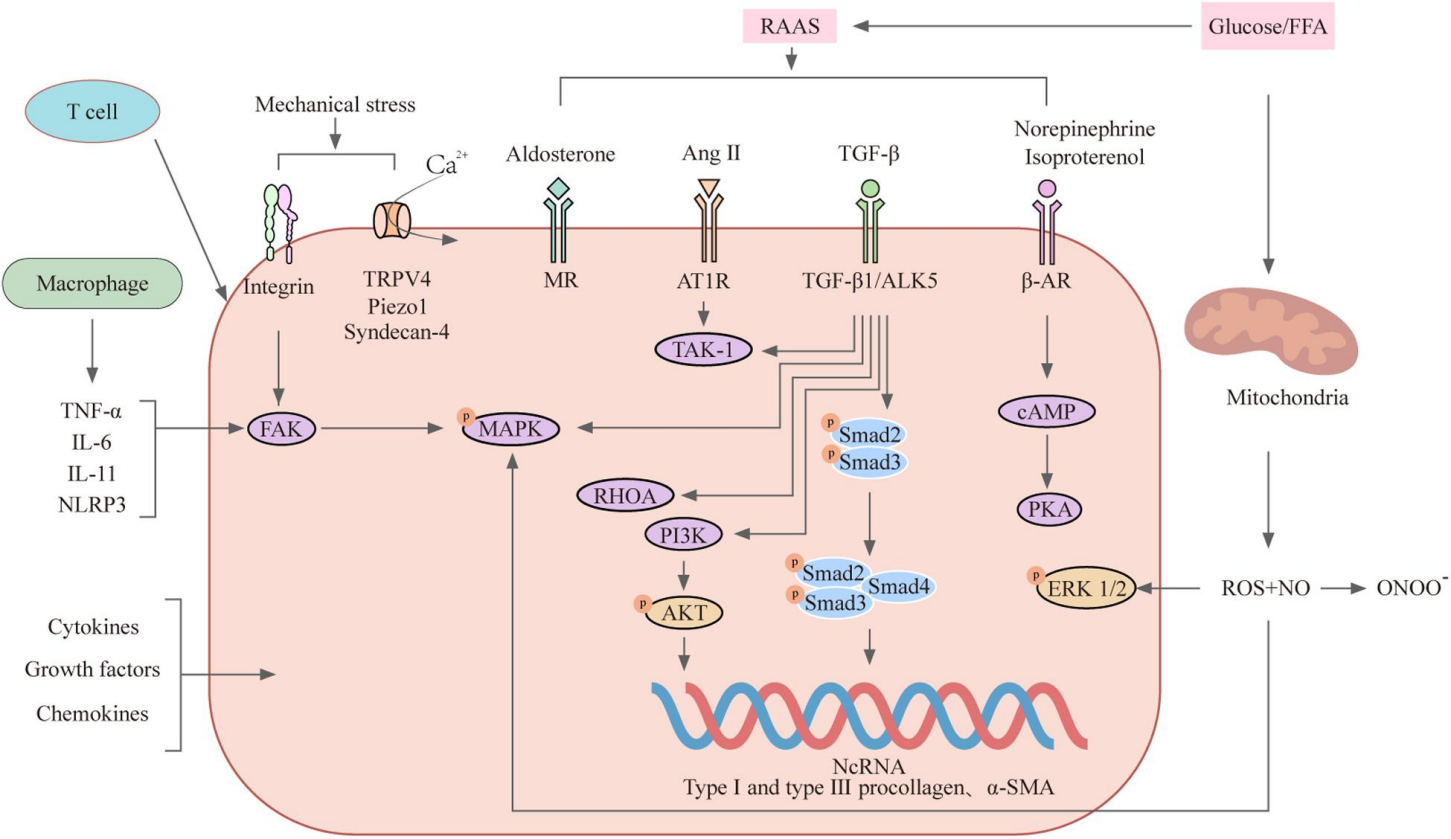
Major signalling pathways involved in myocardial fibrosis.

Within this framework, the following sections outline the major molecular mechanisms underlying myocardial fibrosis, with emphasis on their interactions, regulatory features, and implications for antifibrotic therapy (Figure 1).

This schematic illustrates the interconnected molecular pathways that collectively drive myocardial fibrosis. Mechanical stress is sensed by cardiac fibroblasts through integrins and mechanosensitive ion channels, including transient receptor potential vanilloid 4 (TRPV4) and Piezo1, leading to Ca^2+^ influx and activation of focal adhesion kinase (FAK), RHOA, and mitogen-activated protein kinase (MAPK) signaling cascades. Neurohumoral activation, particularly of the renin–angiotensin–aldosterone system (RAAS), promotes fibroblast activation and myofibroblast differentiation primarily through angiotensin II (Ang II)–induced transforming growth factor-β (TGF-β) signaling, involving both canonical Smad-dependent and non-canonical pathways. Metabolic abnormalities, including elevated levels of glucose and free fatty acids (FFAs), contribute to mitochondrial dysfunction and reactive oxygen species (ROS) overproduction, which amplify profibrotic signaling. Inflammatory responses mediated by immune cells (e.g., macrophages and T cells) further enhance fibrosis through the release of cytokines and chemokines that reinforce shared downstream pathways. Oxidative stress acts as a common amplifier of fibrotic signaling by modulating redox-sensitive pathways, while non-coding RNAs (ncRNAs), including microRNAs, long non-coding RNAs, and circular RNAs, fine-tune profibrotic gene expression at the post-transcriptional level. These convergent signaling networks ultimately disrupt extracellular matrix (ECM) homeostasis by altering the balance between matrix metalloproteinases (MMPs) and tissue inhibitors of metalloproteinases (TIMPs), leading to excessive collagen deposition, increased myocardial stiffness, and impaired cardiac function. Arrows indicate activation or amplification.

### 2.1 Mechanical stress

Mechanical stress is a primary initiating signal in myocardial fibrosis. During the fibrotic process, the continuously changing mechanical properties of the tissue can significantly influence the progression of fibrosis. Under both physiological and pathological conditions (such as hypertension and aortic stenosis), the heart is subjected to various mechanical forces, including contraction from rhythmic beating, shear stress generated by blood flow, and tension resulting from tissue stiffness. Mechanical stress can trigger Ca^2+^ influx via integrins (17, 18) and mechanosensitive ion channels, such as transient receptor potential vanilloid 4 (TRPV4) (19, 20), Piezo1 channels (18, 21, 22), and syndecan-4, thereby activating cardiac fibroblasts (CFs). This process is mediated by the focal adhesion kinase (FAK) (23), RHOA (24, 25), and mitogen-activated protein kinase (MAPK) pathways (26). These pathways promote fibroblast proliferation, cytoskeletal reorganization, and myofibroblast differentiation, thereby priming the myocardium for fibrotic remodeling.

Notably, the profibrotic effects of mechanical stress are preferentially observed in specific fibroblast subpopulations, highlighting the cell-state–dependent nature of mechanotransduction in myocardial fibrosis.

### 2.2 Neurohumoral regulation

Neurohumoral activation, particularly of the RAAS, represents a central driver of myocardial fibrosis. The overactivation of RAAS and the sympathetic nervous system drives fibrosis through specific receptors and downstream signaling pathways. Among these, angiotensin II (Ang II) is widely recognized as an important bioactive substance that promotes myocardial fibrosis; it can activate the proliferation of cardiac fibroblasts and their differentiation into myofibroblasts via the type 1 angiotensin II receptor (AT1R), thereby increasing the expression and secretion of collagen and growth factors (27). In animal experiments involving Ang II infusion, the heart produces endothelin-1 (ET-1) (28) and transforming growth factor-β1 (TGF-β1) (29). TGF-β1 is produced by various cells including immune cells, endothelial cells, cardiomyocytes, and fibroblasts (30); it interacts with the type 1 TGF-β receptor (TGF-βR1, also known as ALK5) and activin receptor-like kinase 4 (ALK4) on the cell membrane, leading to the phosphorylation of Smad2 and Smad3, which then form a complex with Smad4 that translocates to the nucleus, promoting the transcription of fibrotic genes, including those encoding collagen I, collagen III, and α-smooth muscle actin (α-SMA) (31). Aldosterone can also promote myocardial fibrosis through signaling via the mineralocorticoid receptor (32). In addition to driving the recruitment and infiltration of macrophages that induce cardiomyocytes to release pro-fibrotic mediators (33–35), aldosterone can also directly stimulate fibroblast proliferation and collagen production (36). Multiple experimental studies have found that norepinephrine and isoproterenol induce myocardial fibrosis by activating the β1-AR/cAMP/PKA signaling pathway through binding with β*-*adrenergic receptors (β-AR) (37–39).

### 2.3 Metabolic abnormalities

Metabolic abnormalities (such as diabetes, obesity, and insulin resistance) induce myocardial fibrosis by reshaping cellular energy metabolism and promoting a profibrotic microenvironment. Hyperglycemia (40) and lipotoxicity induce mitochondrial dysfunction (41) and oxidative stress, while altered glycolysis (42) and lactate (43) accumulation enhance collagen synthesis and stabilization. These metabolic cues amplify fibrotic signaling primarily by reinforcing shared downstream pathways, including TGF-β–dependent transcriptional programs and redox-sensitive signaling cascades (44, 45).

### 2.4 Inflammatory response

Inflammatory responses act as critical amplifiers of myocardial fibrosis through immune cell–fibroblast interactions. An increasing number of studies indicate that myocardial fibrosis is closely associated with inflammatory responses. The role of macrophages in post-myocardial infarction (MI) fibrosis has been extensively studied (46). Under non-ischemic conditions, patients with diastolic dysfunction exhibit an increased number of cardiac macrophages, which activate fibroblasts by secreting TNF-α, IL-6, and IL-11, thereby promoting collagen deposition (47, 48).

Other immune cells, such as T cells, also play a role in the process of myocardial fibrosis. T cells regulate cardiac fibroblasts (CFs) and MMP activity in cardiac fibrosis and hypertensive conditions (49). In a pressure-overload-induced heart failure mouse model, the recruitment of T cells to the myocardium exacerbates myocardial fibrosis, whereas T cell-deficient mice exhibit reduced fibrosis in response to pressure overload (50, 51). Reports indicate that eight weeks after coronary artery ligation, T cell subpopulations expand in the mouse myocardium, inducing cardiac hypertrophy accompanied by fibrosis (52). T cells contribute to myocardial fibrosis through direct cell-cell interactions mediated by integrin α4 and by activating cardiac fibroblasts via the release of IFN-γ.

Numerous studies have shown that various cytokines, growth factors, and chemokines are involved in the process of myocardial fibrosis. Cytokines such as IL-1, IL-4, IL-6, IL-10, and IL-13 can regulate the phenotypic transformation of cardiac fibroblasts into ECM-synthesizing or MMP-secreting cells (53). Hypoxia-induced mitogenic factor (HIMF) is a secreted pro-inflammatory cytokine, and IL-6, as a downstream signal of HIMF, mediates myocardial fibrosis by activating the MAPK and CaMKII-STAT3 pathways (54). CC-chemokine ligand 2 (CCL2) can stimulate fibroblast proliferation, leading to adverse myocardial remodeling (46). Additionally, as fibroblasts express various DAMP receptors, including Toll-like receptors, NOD-like receptors, IL-1 receptor type 1, and RAGE, cardiac fibroblasts can sense DAMPs released during inflammatory responses (55).

Recent studies have found that the NLRP3 inflammasome is activated in various myocardial cells, including fibroblasts, cardiomyocytes, and injured macrophages, leading to myocardial fibrosis (56). NLRP3 inflammasome-associated proteins are more highly expressed in myocardial fibrosis models (57), suggesting that NLRP3 signaling is enhanced following the induction of myocardial fibrosis (58, 59).

### 2.5 Oxidative stress (OS)

The imbalance between reactive oxygen species (ROS) production and the antioxidant defense system is referred to as OS. OS represents a common downstream mediator linking mechanical, metabolic, and inflammatory stimuli to myocardial fibrosis (60, 61). Excessive ROS production disrupts redox homeostasis, promotes fibroblast activation, and potentiates profibrotic signaling pathways (62, 63). Importantly, oxidative stress does not initiate fibrosis in isolation but acts to intensify and prolong fibrotic responses triggered by upstream pathological stimuli.

### 2.6 Non-coding RNAs (ncRNAs)

NcRNAs function as fine-tuners of myocardial fibrotic signaling by modulating the expression and activity of key profibrotic mediators. Increasing evidence suggests that ncRNAs, including microRNAs (miRNAs), long non-coding RNAs (lncRNAs), and circular RNAs (circRNAs), play a crucial role in the development and progression of myocardial fibrosis. Currently, miRNAs that may be involved in the regulation of fibrosis include miR-1, miR-15, miR-21, miR-22, miR-25, miR-29, miR-30, miR-34, miR-101, miR-122, miR-133, miR-154, miR-185, miR-208, miR-221, miR-495, and miR-590 (64–68). These RNAs do not operate independently as separate pathways. Instead, they regulate the existing signaling networks at the post-transcriptional level, including the TGF-β, MAPK, and oxidative stress-related pathways (69–72), thereby influencing the degree and persistence of fibrosis remodeling.

### 2.7 ECM imbalance

Extracellular matrix imbalance represents the final structural manifestation of myocardial fibrosis. ECM maintains a dynamic balance, and the matrix metalloproteinases (MMPs)/tissue inhibitors of metalloproteinases (TIMPs) system is the primary regulator of this balance (73). MMP-1, MMP-8, and MMP-13 degrade type I, II, and III collagen, thereby inhibiting fibrosis formation. MMP-2 and MMP-9 also process multiple collagen types, including type I, IV, and V, and MMP-2 can additionally cleave type III collagen. MT1-MMP can cleave various ECM proteins, including fibronectin, laminin-1, and type I collagen (73). The insufficient expression and activity of MMPs, particularly MMP-1, along with the excessive expression of TIMPs, are believed to be associated with the development of myocardial fibrosis (74).

However, the existing evidence on fibrotic myocardium in humans is contradictory. For example, compared to patients undergoing off-pump coronary artery bypass grafting, those with severe aortic valve stenosis and myocardial fibrosis exhibit increased myocardial TIMP1 and TIMP2 expression, while MMP1 mRNA levels remain unchanged (75). In contrast, compared to healthy individuals, patients with hypertensive heart disease and heart failure with preserved ejection fraction (HFpEF) show no changes in myocardial MMP1 and TIMP1 levels, whereas those with heart failure with reduced ejection fraction (HFrEF) of the same etiology exhibit an increased MMP1-to-TIMP1 ratio (76). This may be due to excessive degradation of the submembranous collagen scaffold, facilitating myocardial cell slippage and resulting in the loss of synchronized and coordinated myocardial contraction, potentially contributing to left ventricular dilation and impaired systolic function in hypertensive patients presenting with HFrEF and myocardial fibrosis.

## 3 Existing and potential novel therapies

Given that our understanding of myocardial fibrosis (MF) remains incomplete, and our knowledge of the etiology, timing, and mechanistic heterogeneity of fibrosis formation is still insufficient, there are currently no specific anti-MF drugs routinely used in clinical practice. However, some existing heart failure (HF) treatments have shown promising effects on MF. Additionally, certain non-cardiac drugs may have potential antifibrotic therapeutic effects on MF. In recent years, new strategies and drugs targeting profibrotic factors and pathways have been under development (Table 1).

**Table 1:** Current and emerging therapies for myocardial fibrosis.

Drug Category/Therapeutic Strategy	Representative Drugs/Targets	Mechanism of Action and Anti-Fibrotic Effects
RAAS Inhibitors	ACEI, ARB	Exert anti-fibrotic effects through the RAAS system (77, 78)
Aldosterone Receptor Antagonists	Spironolactone	Reduces collagen synthesis (77, 78)
Selective Pacemaker Current Inhibitor	Ivabradine	Inhibits CF proliferation and activation via JNK and p38-MAPK pathways (79)
Beta-Blocker + Pacemaker Current Inhibitor	Carvedilol + Ivabradine	Improves left ventricular diastolic function and survival in cirrhosis patients (80)
Carbonic Anhydrase Inhibitor	Acetazolamide	Reduces sodium reabsorption in the proximal renal tubule, relieving congestion (81, 82)
Anti-Fibrotic Drug	Pirfenidone	Inhibits collagen synthesis by reducing pro-fibrotic and pro-inflammatory cytokine expression (83)
Angiotensin Receptor-Neprilysin Inhibitor	Sacubitril/Valsartan	Reduces fibrosis by lowering MMP-1 and soluble ST2 tissue inhibitor levels (84)
SGLT2 Inhibitors	—	Beyond glucose-lowering effects, reduces cardiovascular death and hospitalization risk in HF patients, involving myocardial fibrosis improvement (10, 85)
Statins	Statins	In addition to lipid-lowering, reduces inflammation and fibrosis (86)
Soluble Guanylate Cyclase (sGC) Stimulator	Vericiguat	Directly stimulates sGC activity, reduces cGMP levels, recommended in 2023 heart failure guidelines, shown to have anti-fibrotic effects (87)
CTGF Inhibitor	Pamrevlumab	Reduces myocardial type I collagen production by inhibiting CTGF activity (88, 89)
TGF-β Inhibitor	—	Direct inhibition of TGF-β may lead to left ventricular dilation and increased mortality risk (90)
Non-Coding RNA (ncRNA) Targeted Therapy	miR-21, miR-29	Therapeutic effects observed in clinical trial stages (91, 92)
Inflammatory Pathway Targeted Therapy	ILs, S100 Proteins	Cardiac fibroblasts respond to inflammatory processes affecting tissue repair (93)
Calpain Inhibitor	BLD-2660	Evaluates changes in ILs and S100A9 protein levels (94)
Immunotherapy (CAR-T Cell Therapy)	FAP-Targeting CAR-T Cells	Targets fibroblast activation protein (FAP), reduces myocardial collagen deposition (95, 96)

ACEI and ARB exert antifibrotic effects through the RAAS system, and aldosterone receptor antagonists such as spironolactone can reduce collagen synthesis (77, 78). Recent studies have found that the selective pacemaker current inhibitor ivabradine exhibits antifibrotic potential in animal models by inhibiting CF proliferation and activation through the JNK and p38-MAPK pathways (79). Researchers have found in clinical treatment that the combination of carvedilol and ivabradine improves left ventricular diastolic function and survival rate in patients with cirrhosis (80). Loop diuretics, commonly used in heart failure patients, have also been shown to impact myocardial fibrosis in several studies. In a multicenter, parallel-group, double-blind, randomized, placebo-controlled trial, acetazolamide (81), a carbonic anhydrase inhibitor that reduces sodium reabsorption in the proximal renal tubule, was associated with a higher rate of decongestion in acute decompensated heart failure patients (82). Pirfenidone, an oral drug used to treat idiopathic pulmonary fibrosis, may inhibit collagen synthesis in tissues by reducing the expression of profibrotic and pro-inflammatory cytokines (83). There is some evidence indicating that pirfenidone has antifibrotic activity in various animal models of heart disease. Angiotensin receptor neprilysin inhibitors, such as sacubitril/valsartan, reduce fibrosis by lowering the levels of MMP-1 and soluble ST2 tissue inhibitors (84). SGLT2 inhibitors (10), in addition to their hypoglycemic effects, show beneficial effects in reducing cardiovascular death and hospitalization risks in heart failure (HF) patients, regardless of whether they have type 2 diabetes. This benefit is thought to be mediated through a series of cardiac actions, including myocardial fibrosis (85). Statins, in addition to their lipid-lowering properties, have multiple potential mechanisms that may benefit the heart, including reducing inflammation and fibrosis (86). Soluble guanylate cyclase (sGC) has become a novel therapeutic target for HF. Vericiguat directly stimulates sGC activity within cells, thereby reducing cGMP levels and the cGMP-dependent signaling pathway. It has become a recommended drug in the 2023 National Heart Failure Guidelines, and recent studies have confirmed its antifibrotic effects (87). However, existing drugs often have issues such as poor targeting and uncertain long-term efficacy, and the specific mechanisms by which some drugs act in humans still require further investigation.

At present, many potential therapeutic approaches are still in the stages of cell models, animal models, and clinical trials. First, connective tissue growth factor (CTGF) is downstream of the TGF-β1 pathway, and treatment with the human monoclonal antibody pamrevlumab, which inhibits its activity, has been shown to reduce myocardial type I collagen production in experimental models (88). In a phase II, double-blind, placebo-controlled trial, pamrevlumab demonstrated good safety and showed antifibrotic efficacy in patients with idiopathic pulmonary fibrosis (89). However, some preclinical studies have shown that direct inhibition of TGF-β in the context of heart failure (HF) can lead to severe left ventricular (LV) dilation and increased mortality (90), indicating that this direct targeted therapy should be approached with caution. Second, some ncRNAs are emerging as potential therapeutic targets for organ fibrosis. Targeted therapies involving miR-21 and miR-29, among others, have shown therapeutic effects in clinical trial stages (91, 92), but challenges regarding their stability and safety remain. Third, treatment strategies targeting inflammation-related pathways can also reduce myocardial fibrosis. Cardiac fibroblasts respond to pro-inflammatory processes related to interleukins and S100 proteins, thereby reducing the heart's ability to respond to injury (93). A phase IIa trial of the calpain inhibitor BLD-2660 will evaluate changes in interleukins and S100A9 protein levels in patients with idiopathic pulmonary fibrosis. However, due to the dual nature of S100A8/A9 as both an inflammatory mediator and an anti-inflammatory agent (94), further research is needed to determine the effective dosage of targeted therapeutics. Fourth, immunotherapy offers a new approach to addressing fibrotic diseases. In a mouse model of hypertensive heart disease, chimeric antigen receptor (CAR) T cells specifically targeting fibroblast activation protein were able to reach the heart, eliminate activated fibroblasts, and reduce collagen deposition (95, 96). This provides support for immunotherapy in myocardial fibrosis, but further research is needed to identify other potential cell types expressing antigens that specifically target cardiac fibroblasts and to investigate their precise roles and mechanisms.

## 4 Conclusion and prospects

Myocardial fibrosis represents a central and potentially modifiable process underlying adverse cardiac remodeling across a wide spectrum of cardiovascular diseases. Accumulating evidence highlights cardiac fibroblast activation, profibrotic signaling pathways such as TGF-β, MAPKs, and inflammatory cascades, as well as metabolic and mechanical stress responses, as key drivers of fibrotic progression. Among emerging therapeutic strategies, interventions targeting fibroblast–immune cell communication, extracellular matrix remodeling, and ncRNA–mediated regulation appear particularly promising for translational application.

Despite these advances, several critical challenges hinder the clinical translation of antifibrotic therapies. Myocardial fibrosis is highly heterogeneous with respect to etiology, disease stage, and spatial distribution, limiting the effectiveness of uniform treatment strategies. Moreover, commonly used animal models fail to fully recapitulate the chronic, multifactorial nature of human cardiac fibrosis. The lack of robust, fibrosis-specific biomarkers further complicates patient stratification and therapeutic monitoring. In addition, many profibrotic pathways play essential roles in tissue repair and homeostasis, raising concerns regarding target specificity and off-target effects.

Future research should focus on precision-based approaches to myocardial fibrosis. Single-cell and spatial omics technologies offer unprecedented opportunities to define disease-specific fibroblast subpopulations and intercellular signaling niches. Artificial intelligence–assisted analyses may facilitate target prioritization, drug repurposing, and rational drug design. Finally, combination therapeutic strategies that simultaneously modulate inflammation, metabolism, and extracellular matrix dynamics may provide superior efficacy compared with single-target interventions. Together, these approaches are expected to advance the development of safe and effective antifibrotic therapies and improve outcomes for patients with cardiovascular disease.
